# Supplementary material for: Enhanced multi-carbon alcohol electroproduction from CO via modulated hydrogen adsorption
Source: Nat Commun. 2020 Jul 23;11:3685. doi: 10.1038/s41467-020-17499-5 (PMC7378828; doi:10.1038/s41467-020-17499-5)
Supplement: Supplementary file 1 — Supplementary Information [file 41467_2020_17499_MOESM1_ESM.pdf]

Supplementary Information for

Enhanced multi-carbon alcohol electroproduction from CO via modulated hydrogen adsorption

Jun Li *et al.*

Supplementary Figures

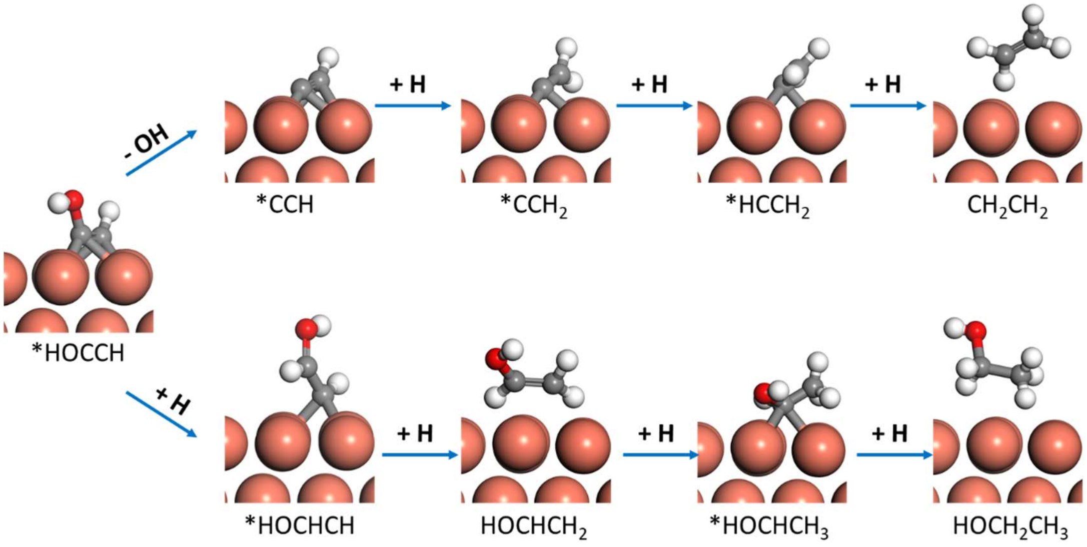

Supplementary Fig. 1. The competing reaction pathway for CO reduction to ethylene (CH_2CH_2) and alcohol (e.g. ethanol, CH_3CH_2OH) proposed by Goddard and co-workers[1,2].

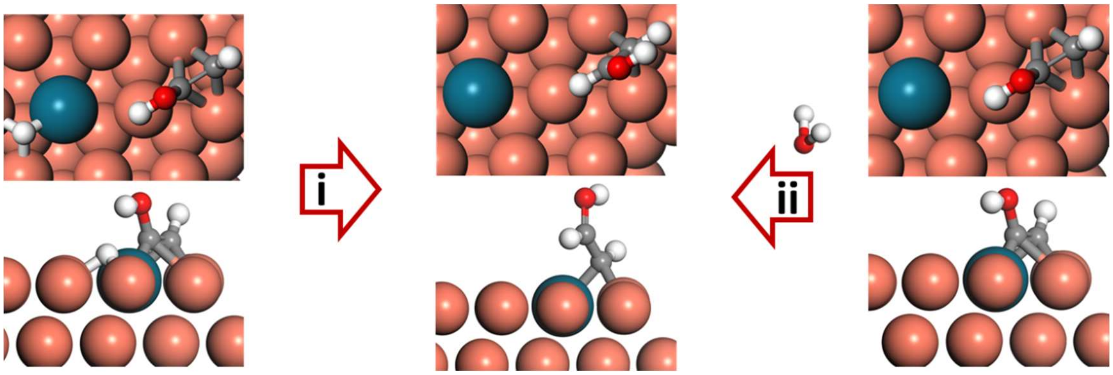

Supplementary Fig. 2. The hydrogenation process of HOCCH* with H sources from (i) adsorbed H* (left) and (ii) H_2O (right).

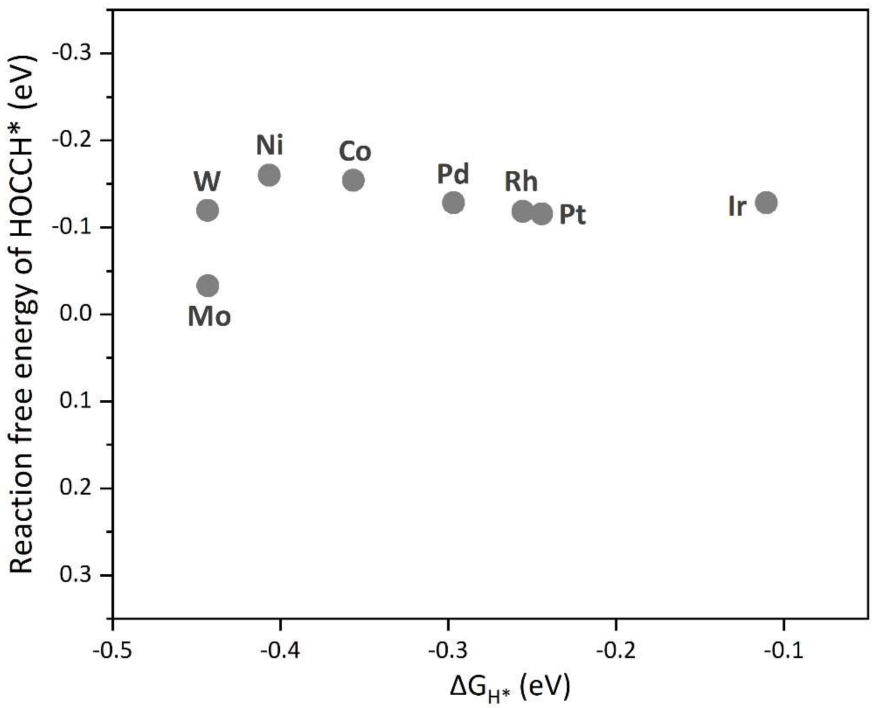

Supplementary Fig. 3. The reaction free energies for the dehydroxylation reaction of HOCCH* on different doped Cu surfaces.

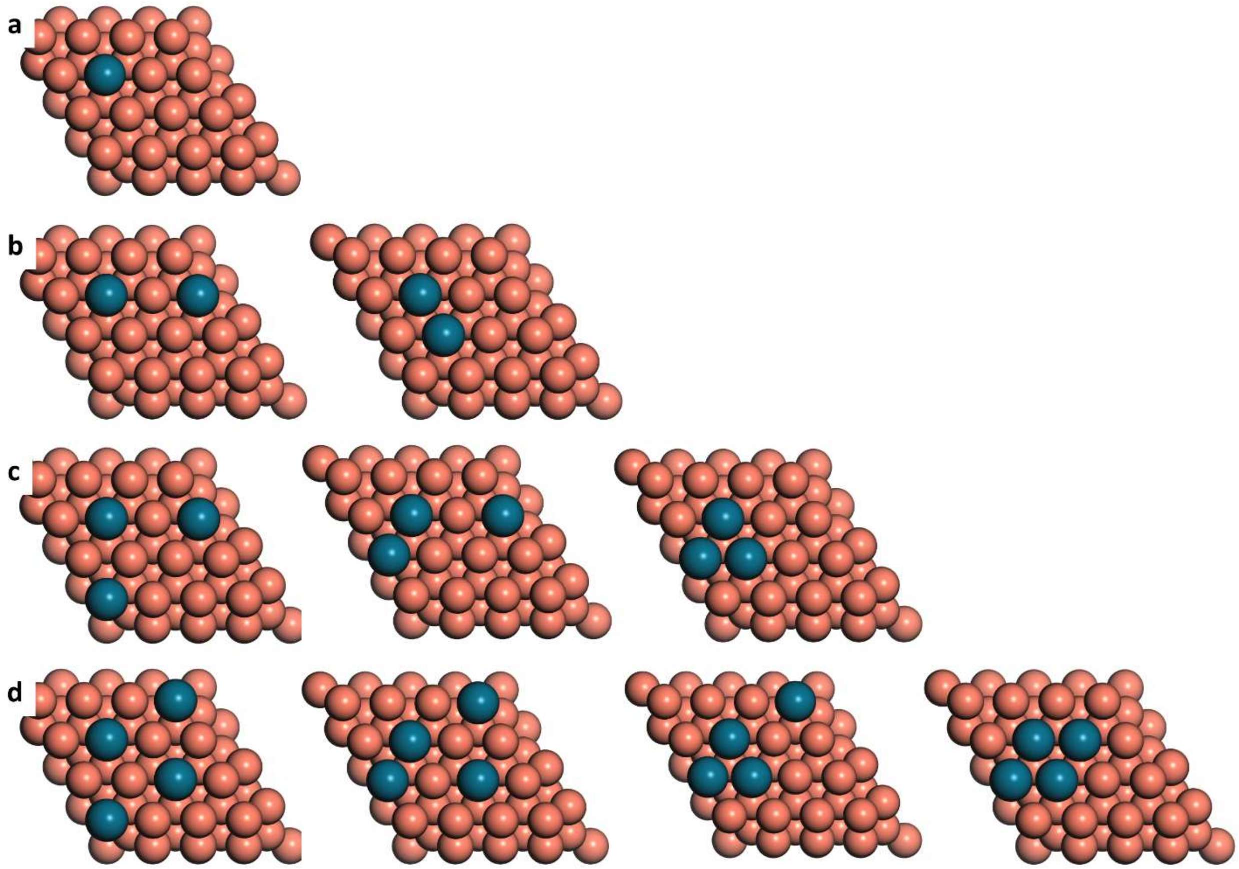

Supplementary Fig. 4. A schematic view of the Pd-doped Cu surfaces. a-d, The Pd-modified Cu(111) surfaces with varying Pd concentrations of 1/16 (**a**), 1/8 (**b**), 3/16 (**c**), 1/4 (**d**), respectively; different distributions of surface Pd dopants are indicated horizontally at each row with the same Pd loading.

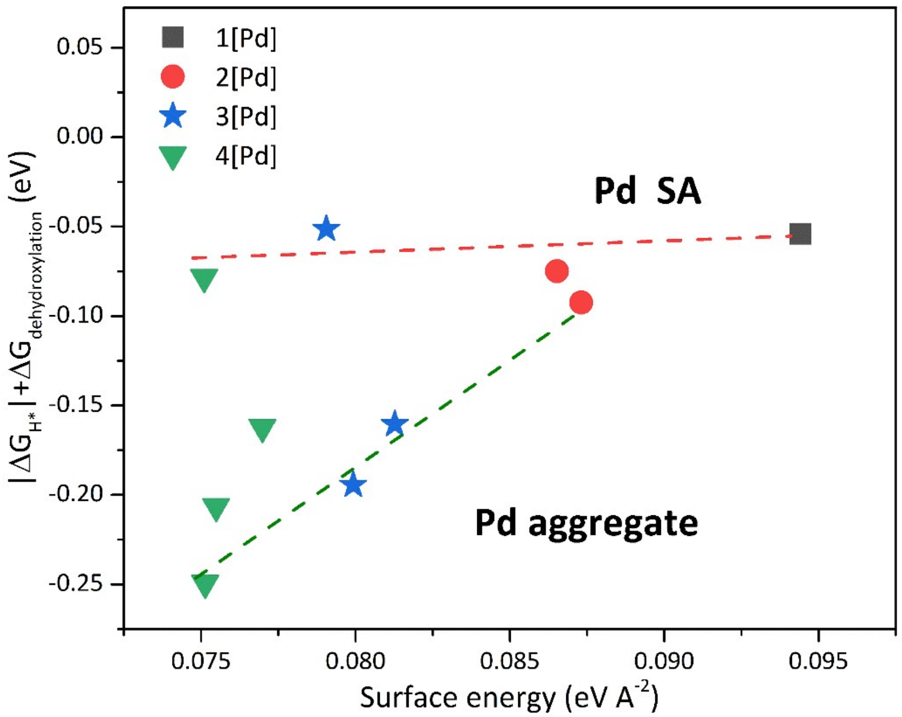

Supplementary Fig. 5. The reaction free energies of hydrogen evolution reaction and dehydroxylation reaction of HOCCH* for different Pd doping configurations, where atomically dispersed Pd configurations have weaker selectivity to these two reactions with high reaction free energies.

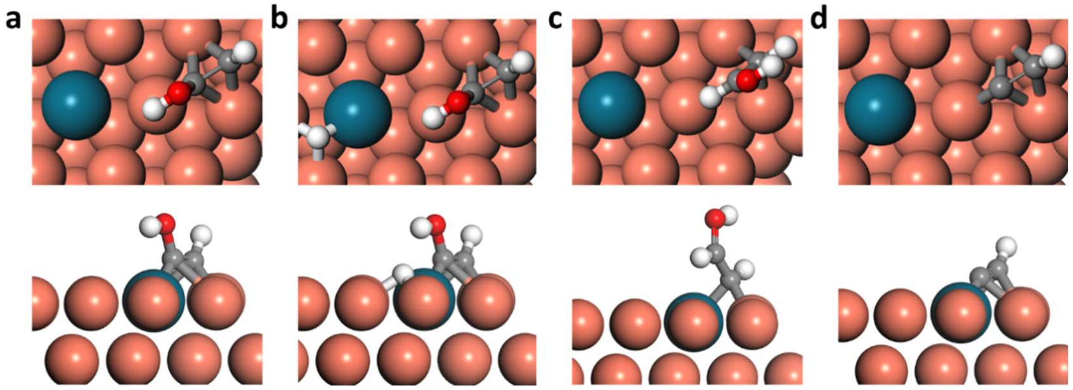

Supplementary Fig. 6. Adsorption configuration of C_2 intermediates at the Pd-doped Cu surface. a-d, The top and side views of absorption configurations for HOCCH* (**a**), HOCCH*+H* (**b**), HOCHCH* (**c**) and CCH* (**d**) on the Pd doped Cu (111) surface.

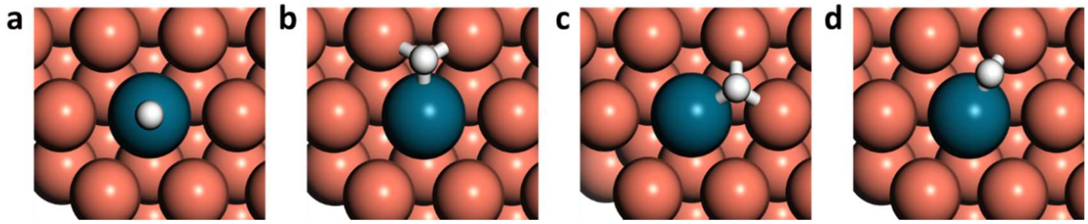

Supplementary Fig. 7. Hydrogen adsorption at the Pd-doped Cu surface. a-d, Top views of H absorptions on the top (**a**), fcc (**b**), hcp (**c**) and bridge (**d**) sites of Pd doped Cu (111) surface.

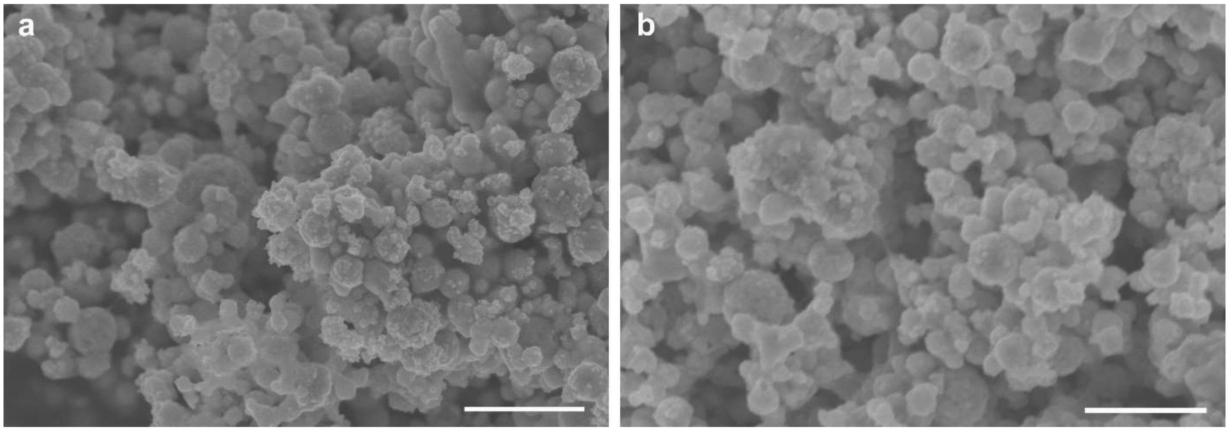

Supplementary Fig. 8. Morphology characterization of different Cu catalysts. a, b, SEM images of bare-Cu (**a**) and Pd-doped Cu (**b**) electrocatalysts after COR at -0.62V vs. RHE in 1 M KOH. The scale bars are 500 nm.

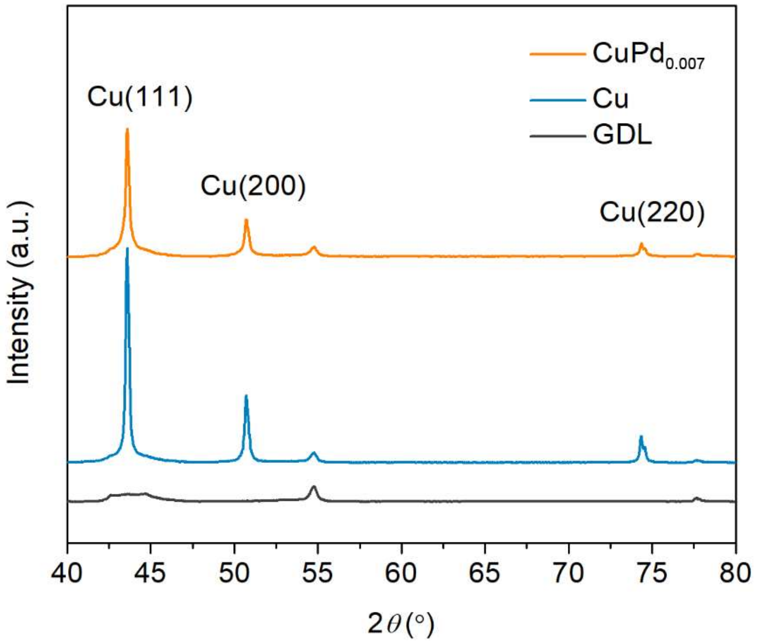

Supplementary Fig. 9. Powder X-ray diffraction spectra of gas diffusion layer (GDL), Cu/GDL and $CuPd_{0.007}$/GDL electrocatalysts after COR at -0.62V vs. RHE in 1 M KOH.

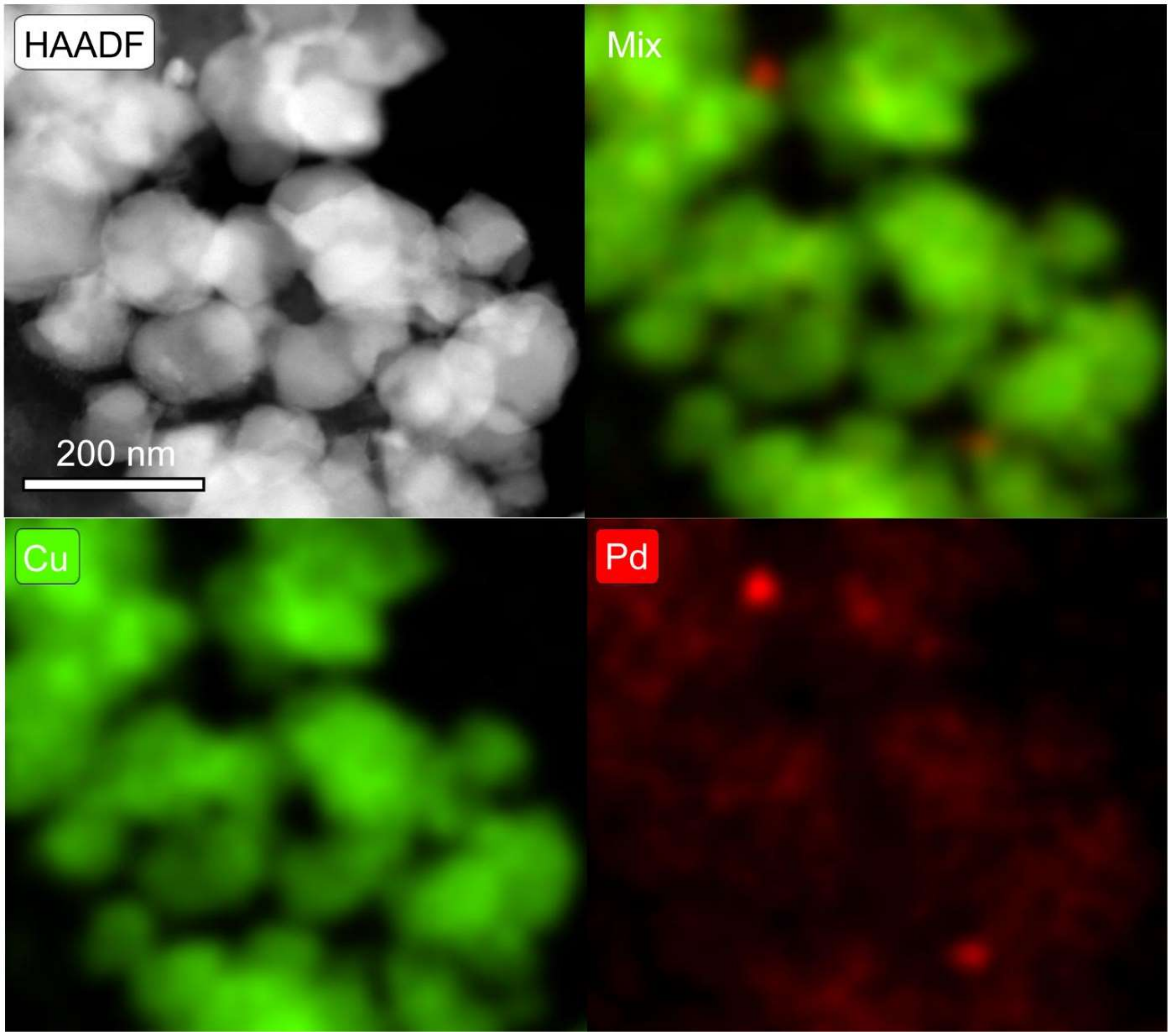

Supplementary Fig. 10. HAADF image and EDX mapping of $CuPd_{0.007}$ electrocatalyst after COR at -0.62V vs. RHE in 1 M KOH.

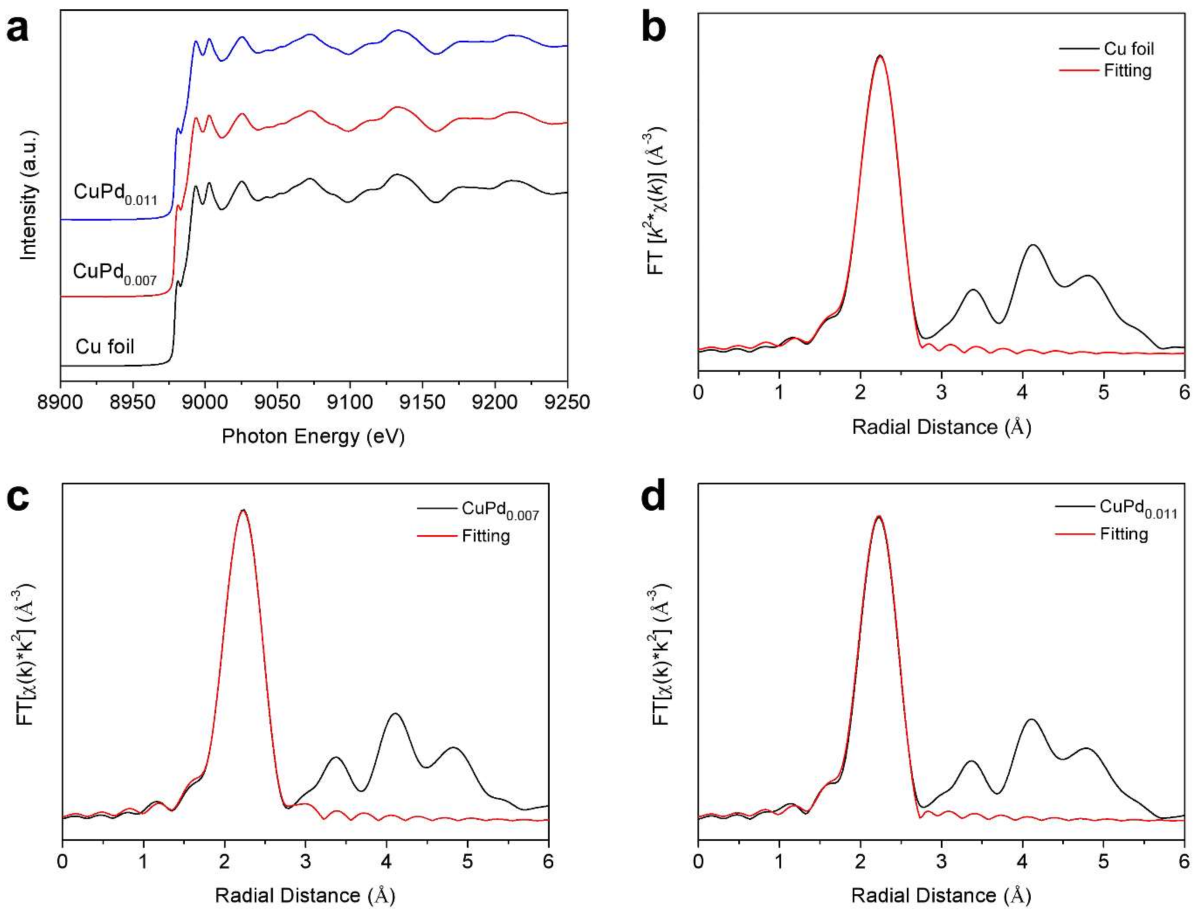

Supplementary Fig. 11. Cu K-edge XAS of different Cu samples. a-d, Operando XANES (**a**) and EXAFS spectra at the Cu K-edge of Cu foil (**b**), $CuPd_{0.007}$ (**c**) and $CuPd_{0.011}$ (**d**) during COR at -0.62V vs. RHE in 1 M KOH.

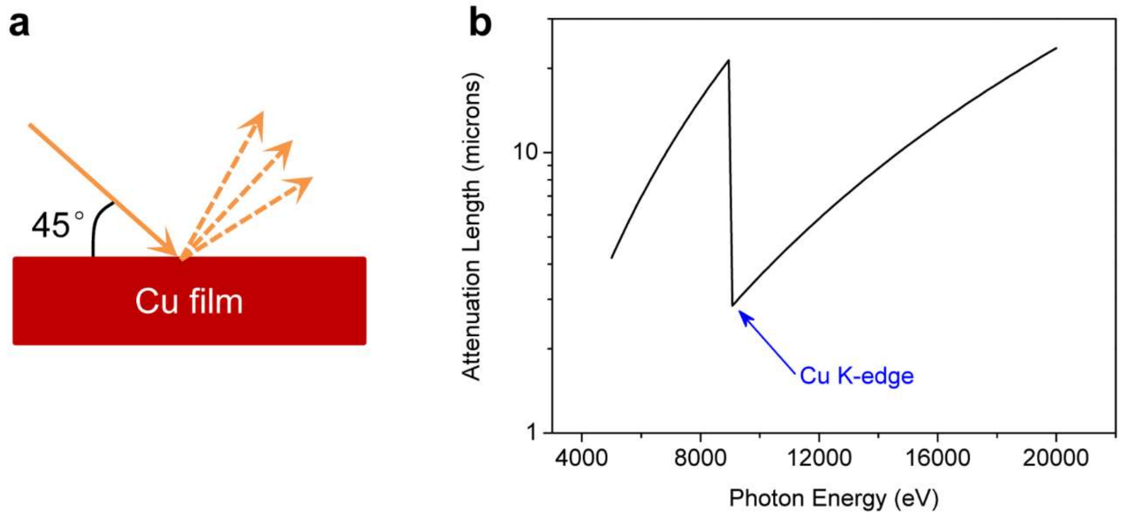

Supplementary Fig. 12. Estimation of XAS probing depth. a, Illustration of XAS measurements with an incidence angle of 45º. **b,** Calculated attenuation length of X-rays with an incidence angle of 45º to a Cu film (density = 8.96 g cm^{-3}) at a photon energy range close to the Cu K-edge (http://henke.lbl.gov/optical_constants/atten2.html).

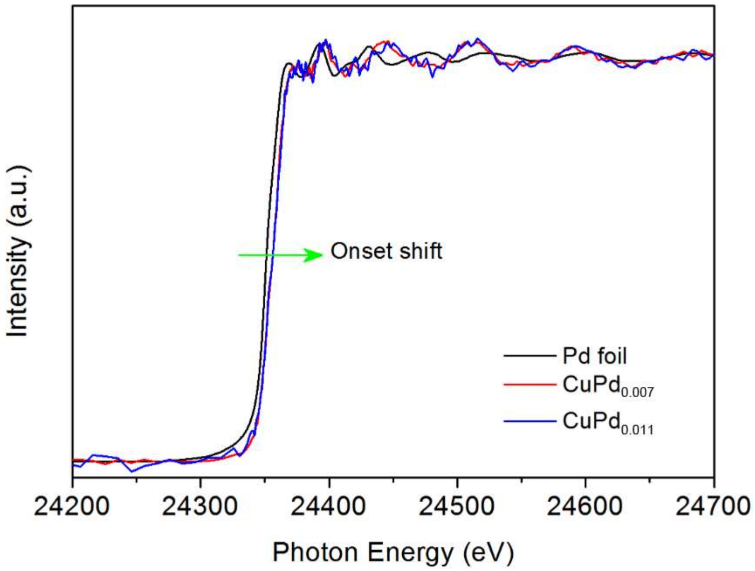

Supplementary Fig. 13. Ex-situ Pd K-edge XAS spectra of Pd foil, $CuPd_{0.007}$ and $CuPd_{0.011}$ after COR at -0.62V vs. RHE in 1 M KOH.

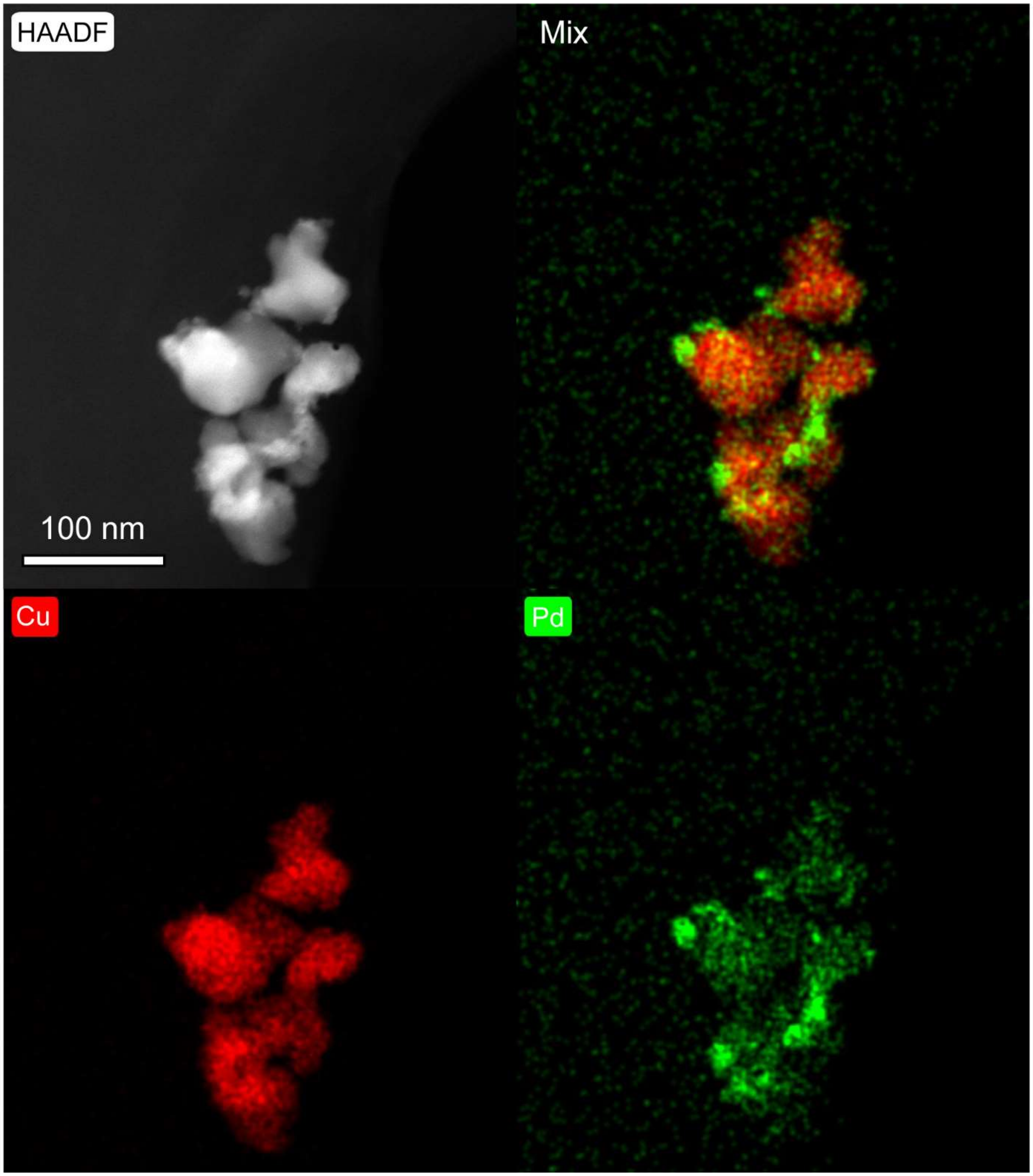

Supplementary Fig. 14. HAADF image and EDX mapping of $CuPd_{0.011}$ electrocatalyst after COR at -0.62V vs. RHE in 1 M KOH.

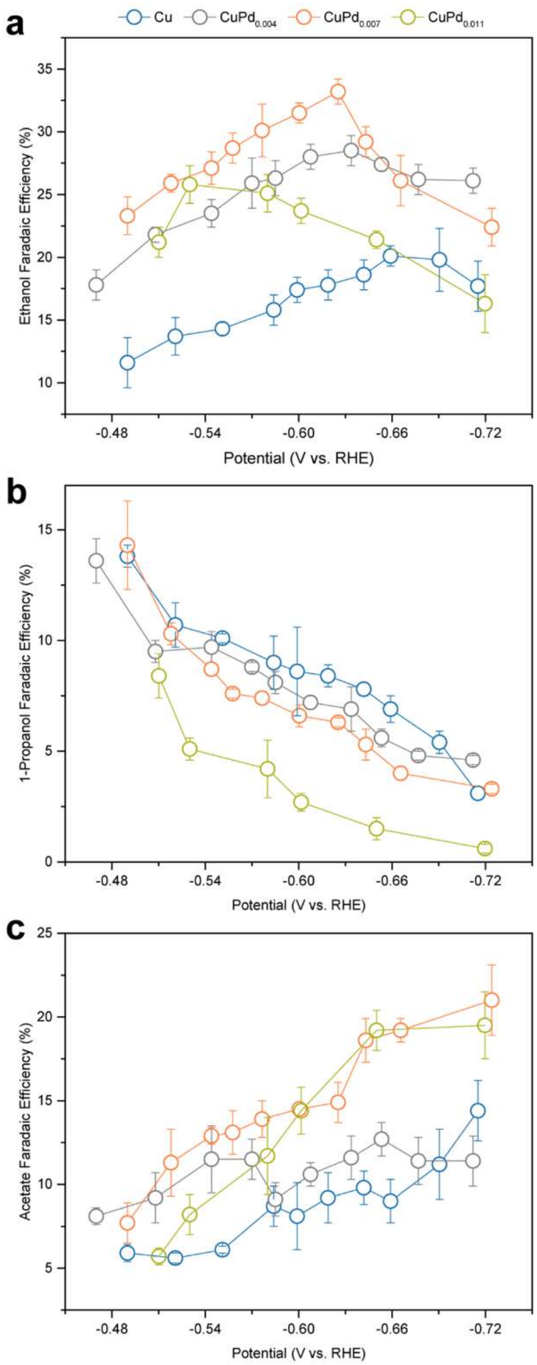

Supplementary Fig. 15. COR selectivity of Pd-doped Cu electrocatalysts. a-c, COR product selectivities (FE, %) towards ethanol (**a**), 1-propanol (**b**) and acetate (**c**) productions on Cu, $CuPd_{0.004}$, $CuPd_{0.007}$ and $CuPd_{0.011}$ catalysts at various applied potentials (vs. RHE) in 1 M KOH. Error bars are means ± SD (n = 3 replicates).

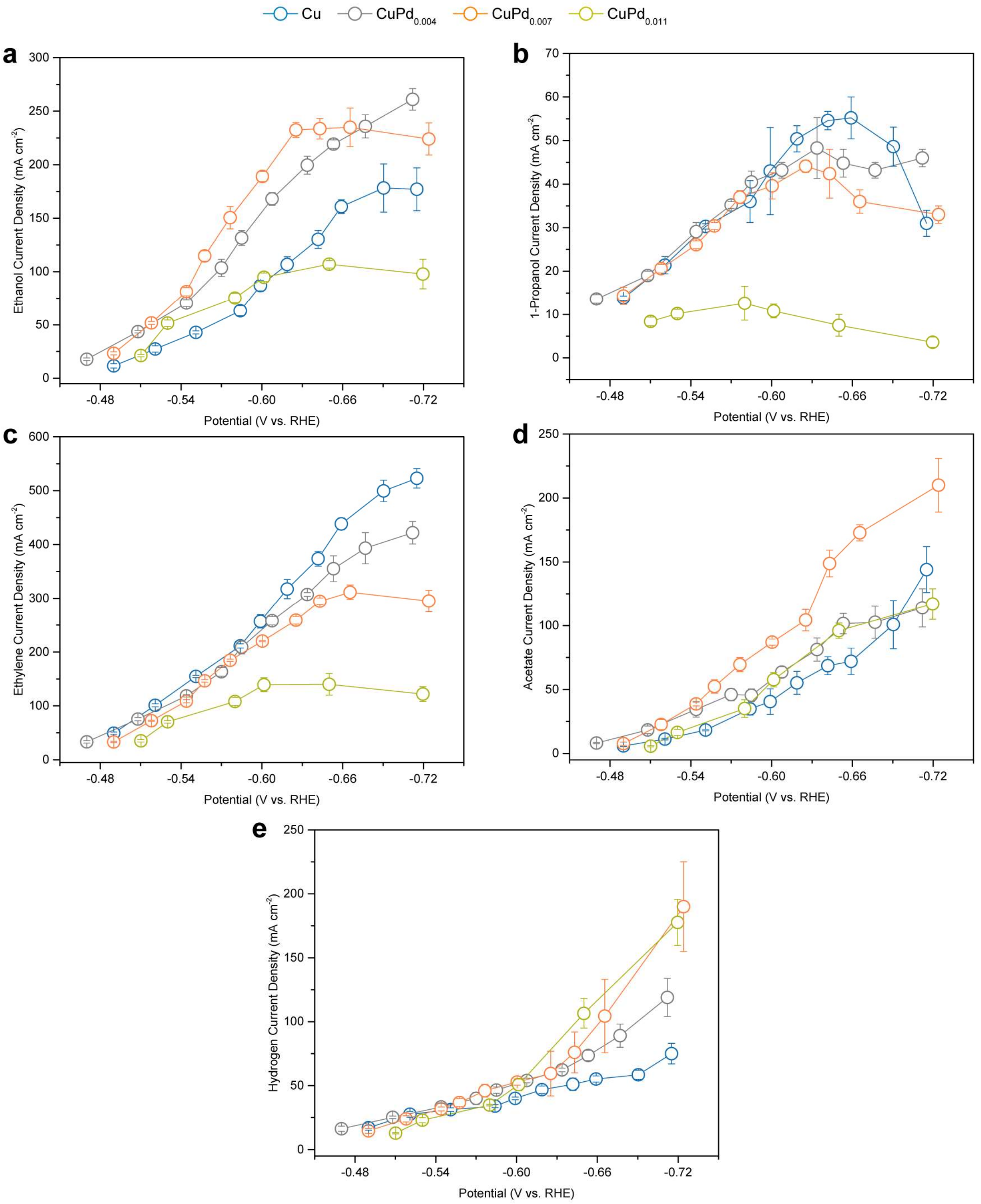

Supplementary Fig. 16. COR activity of Pd-doped Cu electrocatalysts. a-e, COR product activities (partial current densities, mA cm^{-2}) towards ethanol (**a**), 1-propanol (**b**), ethylene (**c**), acetate (**d**) and hydrogen (**e**) productions on Cu, $CuPd_{0.004}$, $CuPd_{0.007}$ and $CuPd_{0.011}$ catalysts at various applied potentials (vs. RHE) in 1 M KOH.

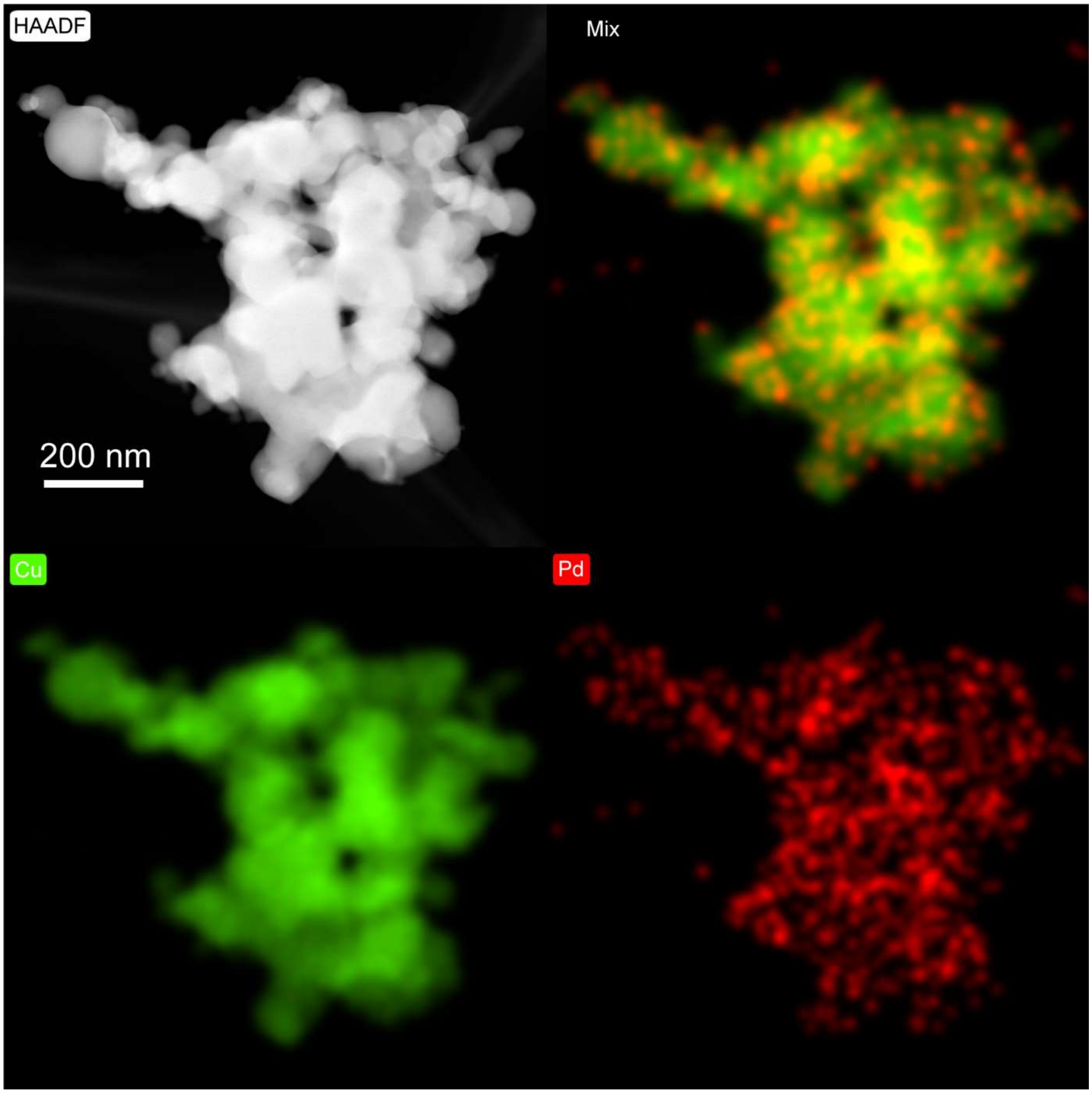

Supplementary Fig. 17. HAADF image and EDX mapping of aggregated $CuPd_{0.008}$ electrocatalyst after COR at -0.63V vs. RHE in 1 M KOH. This catalyst synthesis procedure is similar to the one used to prepare one atomic-level $CuPd_{0.007}$ catalyst (See *Methods*). However, instead of applying intense ultrasonication, the galvanic replacement reaction was initiated at a continuous stirring condition (300 rpm) in room temperature for 45 mins under inert N_2 gas protection.

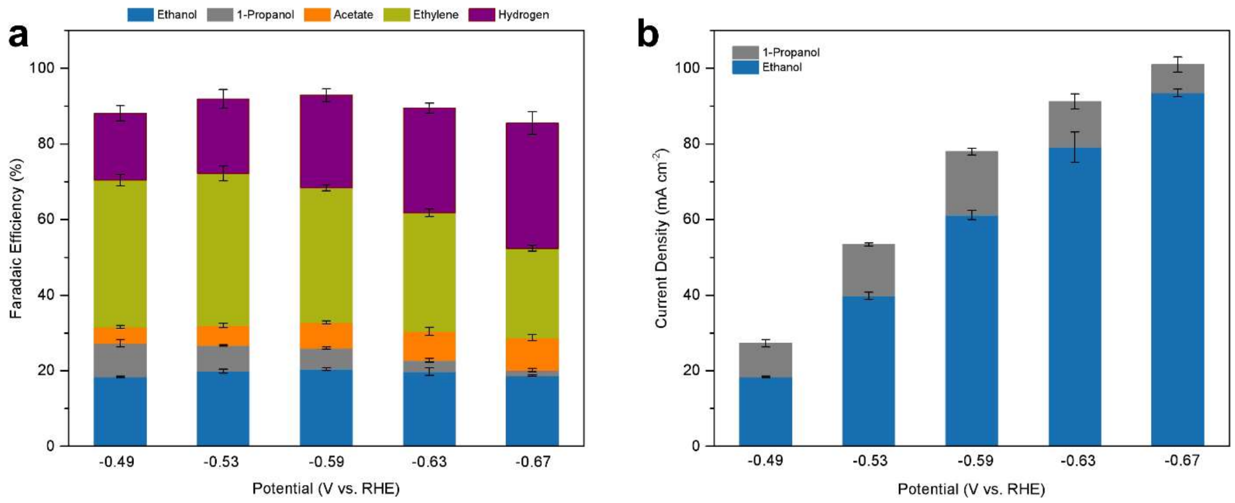

Supplementary Fig. 18. COR performance of aggregate CuPd electrocatalysts. a, b, COR product selectivities (FE, %) (**a**) and alcohol partial current density (mA cm^{-2}) (**b**) of aggregated $CuPd_{0.008}$ catalysts at various applied potentials (vs. RHE) in 1 M KOH. Error bars are means ± SD (n = 3 replicates).

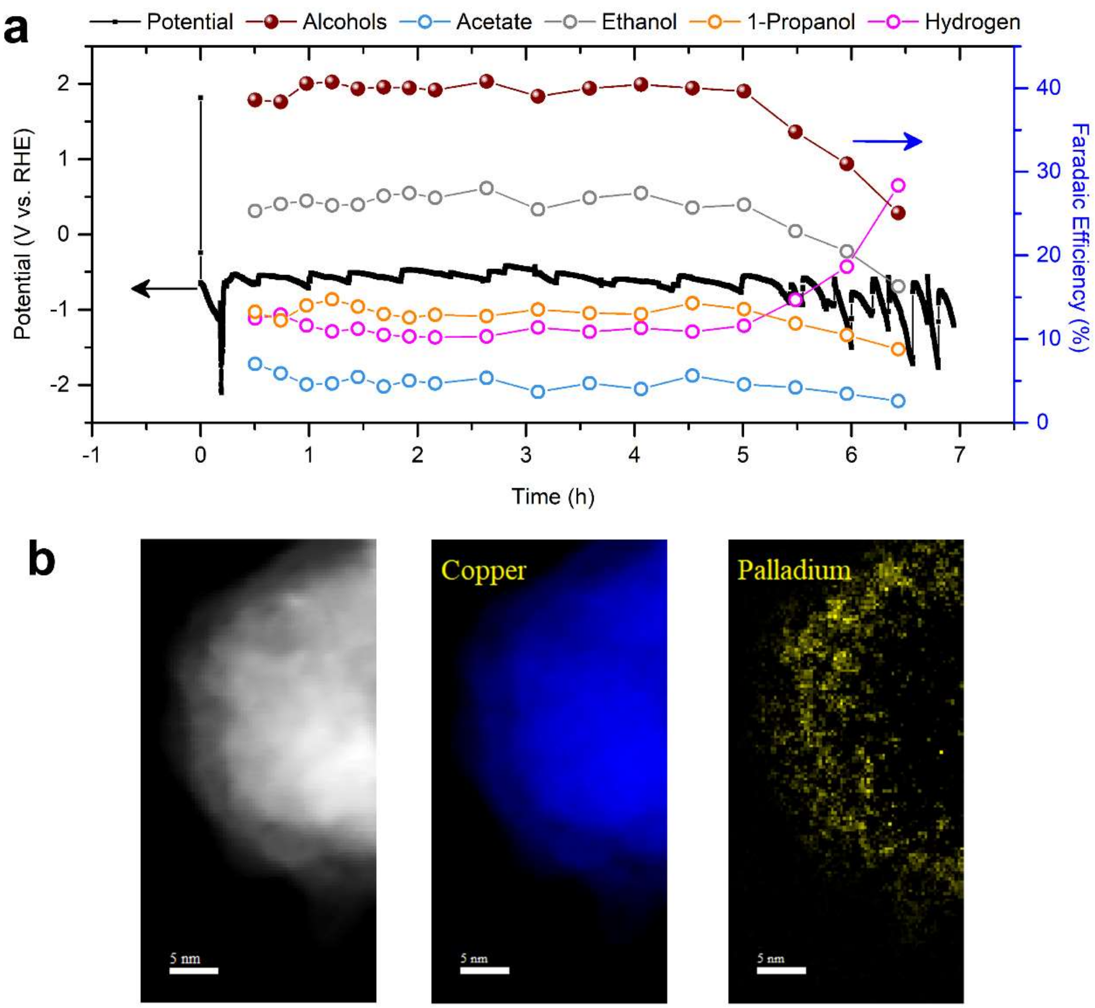

c

Sample ID	Cu (μg mL-1)	Pd (μg mL-1)	Cu:Pd ratio
$CuPd_{0.007}$ (before)	473.9	5.6	1:0.007
$CuPd_{0.007}$ (after)	243.5	1.2	1:0.003

Supplementary Fig. 19. COR stability of Pd-doped Cu electrocatalysts. a, COR performance of the $CuPd_{0.007}$ catalyst loaded on a polytetrafluorethylen membrane gas diffusion layer (pore size of 0.45 μm) over a course of 7 hours operation in 1 M KOH at a constant current density of 100 mA cm^{-2}. **b,** HAADF-STEM images of $CuPd_{0.007}$ after 7 hours COR test. **c,** Elemental composition of Cu and Pd in $CuPd_{0.007}$ before and after 7 hours COR test determined by inductively coupled plasma optical emission spectrometry (ICP-OES).

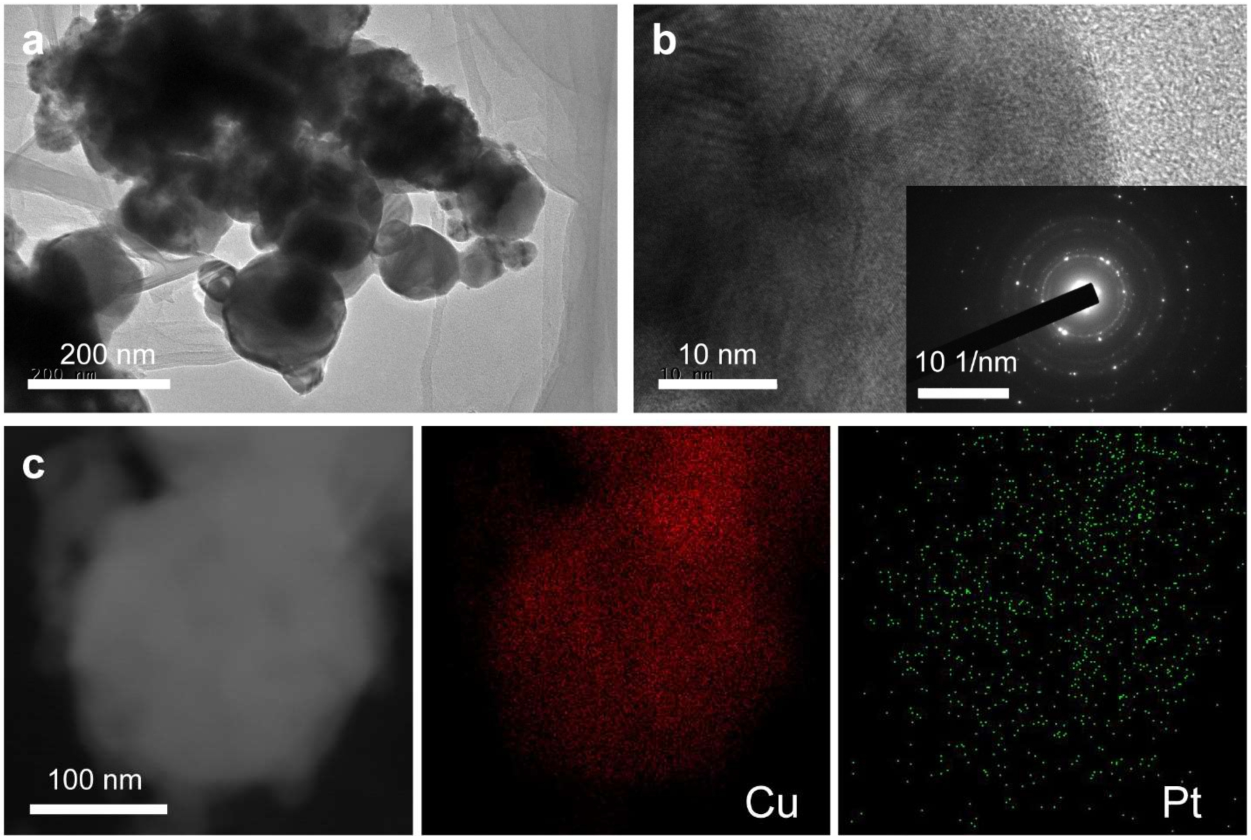

Supplementary Fig. 20. TEM and HAADF-STEM images of Pt-doped Cu ($CuPt_{0.008}$) electrocatalysts after COR at -0.63V vs. RHE in 1 M KOH.

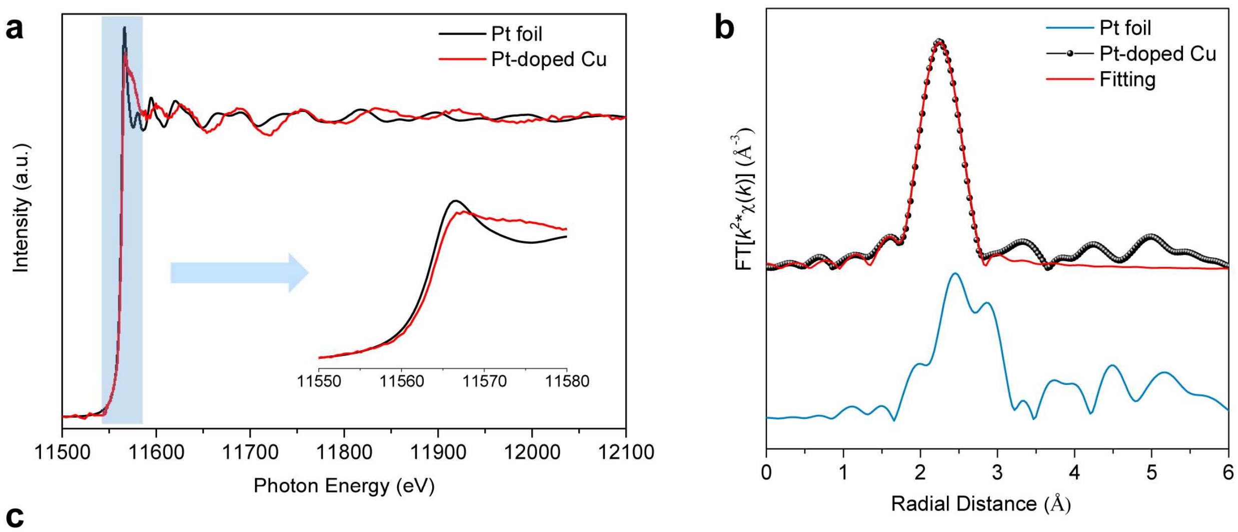

c

Sample	Shell	R (Å)	CN	ΔE_0 (eV)	σ^2 (10^{-3} Å^2)
Pt foil	Pt-Pt	2.77	12	8.11	4.51
Pt-doped Cu	Pt-Cu	2.59	8.2	6.01	6.51

Supplementary Fig. 21. X-ray absorption analysis of Pt-doped Cu electrocatalysts. a, Pt L_3-edge XAS spectra of Pt foil and Pt-doped Cu catalyst. **b,** Pt L_3-edge EXAFS spectra as well as corresponding fitting spectra of Pt-doped Cu catalyst (top) and Pt foil (bottom). **c,** Pt L_3-edge EXAFS simulation results of Pt foil and Pt-doped Cu catalyst. Operando XAS measurement of Pt-doped Cu catalyst was taken during COR at -0.63V vs. RHE in 1 M KOH.

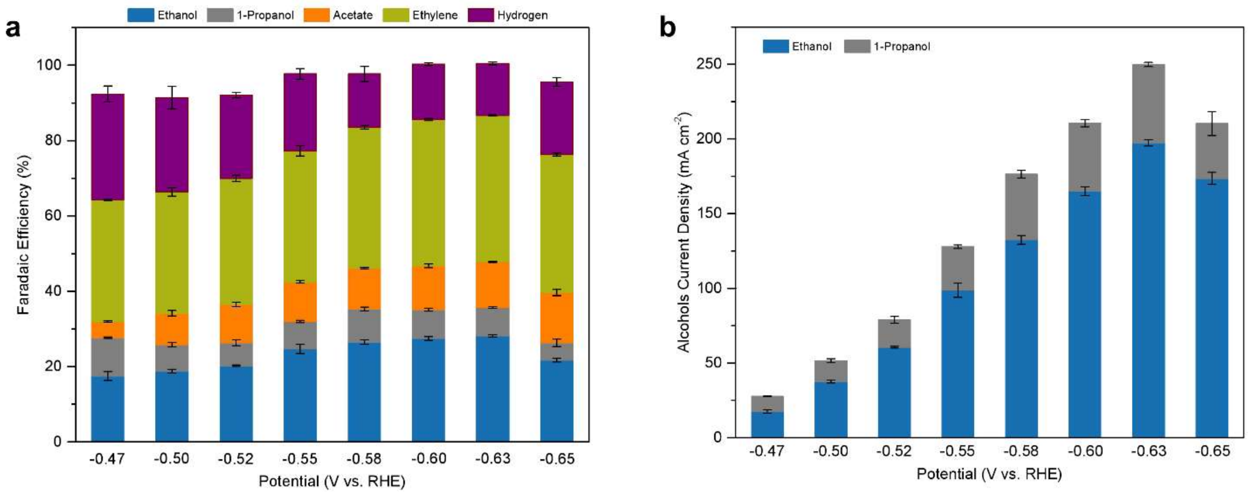

Supplementary Fig. 22. COR performance of Pt-doped Cu electrocatalysts. a, b, COR product selectivities (FE, %) (**a**) and alcohol partial current density (mA cm^{-2}) (**b**) of Pt-doped Cu catalyst at various applied potentials (vs. RHE) in 1 M KOH. Error bars are means ± SD (n = 3 replicates).

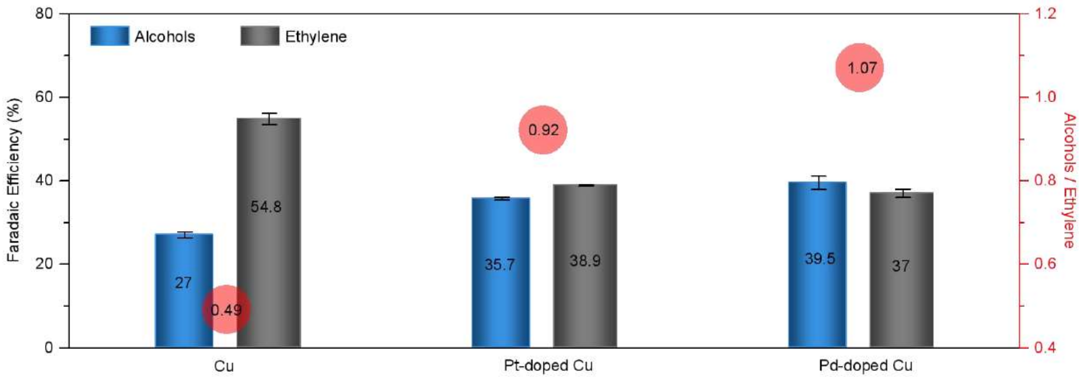

Supplementary Fig. 23. The peak alcohols selectivities & ethylene productions (left) and corresponding $FE_{alcohols}/FE_{ethylene}$ ratio (right) from COR on different catalysts at -0.66 V (Cu), -0.63 V ($CuPt_{0.008}$) and -0.62 V ($CuPd_{0.007}$) vs. RHE. Numbers in the red circles show the corresponding Faradaic efficiency ratio of alcohols to ethylene. Error bars are means ± SD (n = 3 replicates).

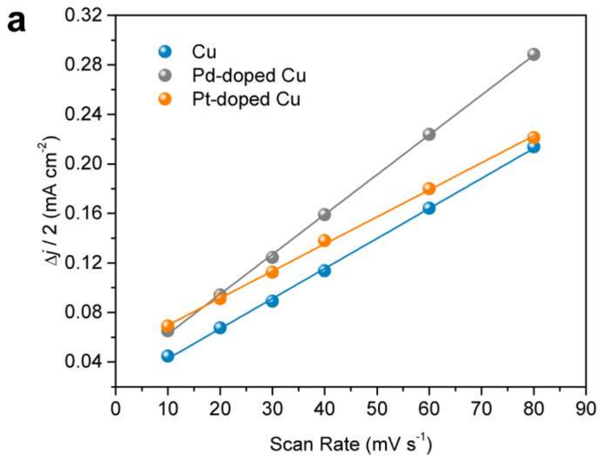

b

Sample	Capacitance	Surface roughness factor
Cu foil	29 μF	1
Cu	2.43 mF	84
Pd-doped Cu	3.22 mF	111
Pt-doped Cu	2.03 mF	70

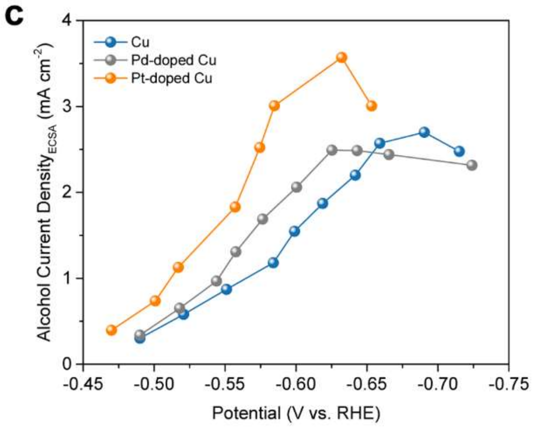

Supplementary Fig. 24. Electrochemical surface area measurement. a, Determination of double-layer capacitance on various catalysts by cyclic voltammograms taken over a range of scan rates. **b,** Tabulated values of capacitance and corresponding surface roughness factor for different catalysts by normalizing the surface roughness factor of Cu foil to 1 (Ref.[3]). **c,** Electrochemical surface area normalized partial currents of alcohols on different catalysts.

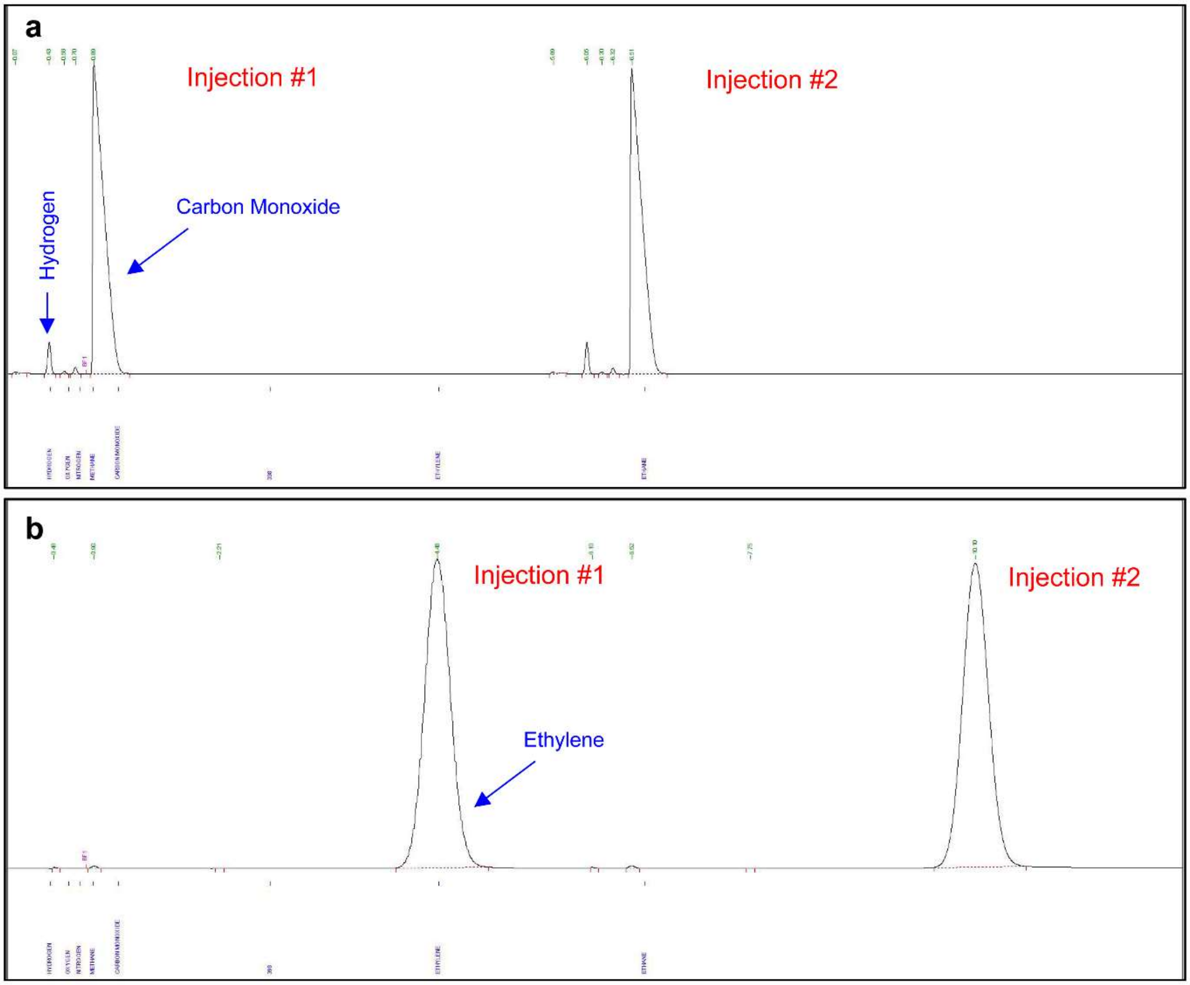

Supplementary Fig. 25. Gas chromatography measurement. a, b, Representative GC traces of COR gaseous products on $CuPd_{0.007}$ catalysts at -0.62 V vs. RHE in 1M KOH from thermal conductivity detector (**a**) and flame ionization detector (**b**) channels.

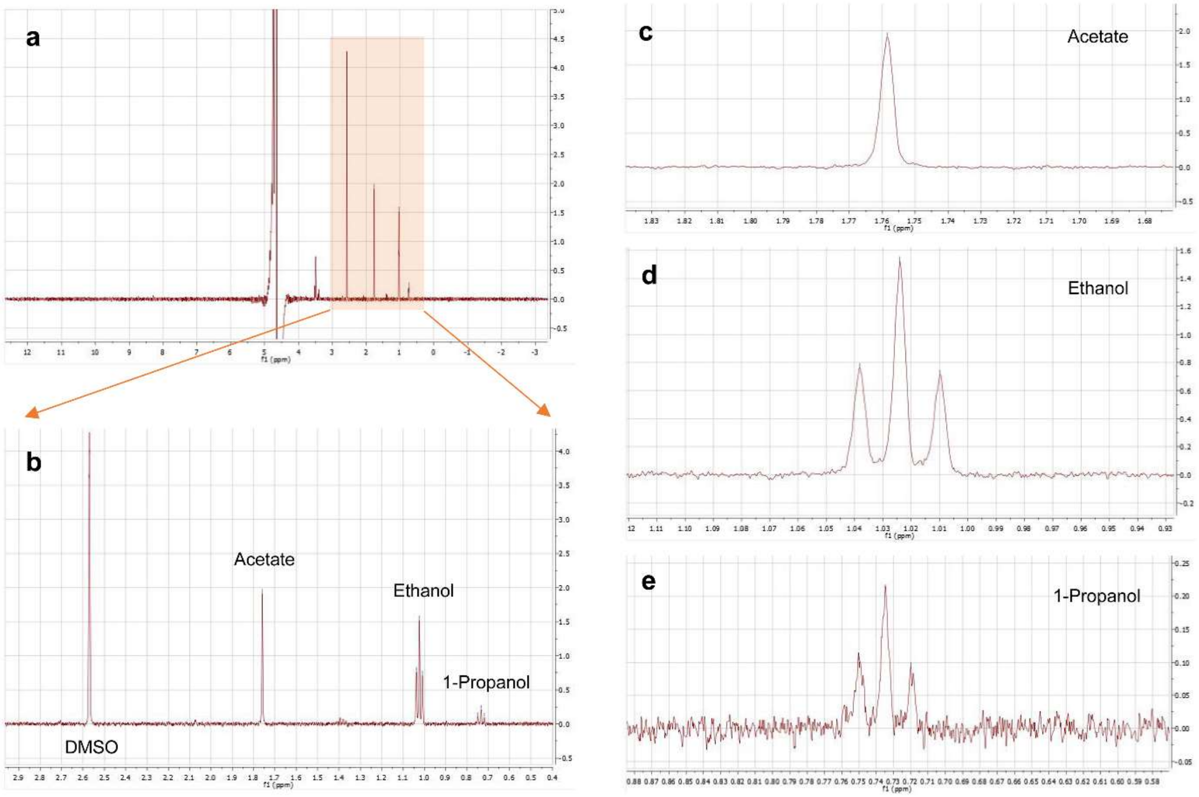

Supplementary Fig. 26. [1]H Nuclear magnetic resonance spectroscopy measurement. a, Representative ^{1}H-NMR spectra of COR liquid products on $CuPd_{0.007}$ catalysts at -0.62 V vs. RHE in 1M KOH. **b,** An enlarged view of area highlighted in (**a**). **c-e,** ^{1}H-NMR spectra of acetate (**c**), ethanol (**d**) and 1-propanol (**e**). DMSO (Dimethyl sulphoxide) was used as an internal for liquid products quantification.

Supplementary Tables

Supplementary Table 1. Total energies (ΔE), zero point energies (ΔZPE), entropy changes (TΔS) and free energies (ΔG) for the hydrogenation reaction of HOCCH* on the Cu surface using different H sources.

H source	ΔE (eV)	ΔZPE (eV)	TΔS (eV)	ΔG (eV)
Adsorbed H*	-0.11	0.15	-	0.04
Water	-0.25	0.17	-0.21	0.13

Supplementary Table 2. Total energies (ΔE), zero point energies (ΔZPE) and free energies (ΔG) for H absorption on different metal surfaces.

Metals	Absorption sites	ΔE (eV)	ΔZPE (eV)	ΔG (eV)
W	bridge	-0.67	0.03	-0.44
	hollow	-0.55	0.00	-0.34
	top	-0.02	-0.02	0.16
Mo	bridge	-0.67	0.02	-0.44
	hollow	-0.61	0.00	-0.40
	top	0.03	-0.04	0.19
Ni	bridge	-0.65	0.04	-0.40
	fcc	-0.65	0.04	-0.41
	hcp	-0.64	0.04	-0.39
	top	-0.05	-0.02	0.13
Co	bridge	-0.60	0.04	-0.36
	fcc	-0.60	0.04	-0.36
	hcp	-0.60	0.04	-0.36
	top	-0.08	-0.02	0.11
Pd	bridge	-0.52	0.02	-0.30
	fcc	-0.52	0.02	-0.30
	hcp	-0.48	0.02	-0.25
	top	-0.02	-0.01	0.17
Rh	bridge	-0.48	0.02	-0.25
	fcc	-0.48	0.02	-0.26
	hcp	-0.48	0.02	-0.25
	top	-0.13	0.00	0.07
Pt	bridge	-0.15	0.01	0.06
	fcc	-0.45	0.00	-0.24
	hcp	-0.41	0.00	-0.21
	top	-0.17	0.04	0.08
Ir	bridge	-0.31	0.00	-0.11
	fcc	-0.35	0.00	-0.14
	hcp	-0.34	0.00	-0.13
	top	-0.42	0.02	-0.19

Supplementary Table 3. Total energies (ΔE), zero point energies (ΔZPE) and free energies (ΔG) for the hydrogenation reaction of HOCCH* with adsorbed H* on different doped Cu surfaces.

Dopant elements	ΔE (eV)	ΔZPE (eV)	ΔG (eV)
W	0.15	0.15	0.30
Mo	0.11	0.15	0.26
Ni	-0.14	0.14	0.00
Co	-0.02	0.14	0.12
Pd	-0.30	0.15	-0.15
Rh	-0.11	0.15	0.04
Pt	-0.25	0.16	-0.09
Ir	-0.01	0.16	0.15

Supplementary Table 4. Total energies (ΔE), zero point energies (ΔZPE) and free energies (ΔG) for the dehydroxylation reaction of HOCCH* on different doped Cu surfaces.

Dopant elements	ΔE (eV)	ΔZPE (eV)	ΔG (eV)
W	0.26	-0.01	-0.12
Mo	0.27	0.08	-0.03
Ni	0.23	-0.01	-0.15
Co	0.22	-0.01	-0.16
Pd	0.26	-0.01	-0.12
Rh	0.25	-0.01	-0.13
Pt	0.27	-0.01	-0.12
Ir	0.25	-0.01	-0.13

Supplementary Table 5. Elemental composition of Cu and Pd in different CuPd catalysts determined by inductively coupled plasma optical emission spectrometry (ICP-OES).

Nominal Cu:Pd ratio	Cu (μg mL^{-1})	Pd (μg mL^{-1})	Experimental Cu:Pd ratio
1:0.005	563.1	3.4	1:0.004
1:0.010 (homogeneous)	473.9	5.6	1:0.007
1:0.010 (aggregate)	885.4	12.4	1:0.008
1:0.015	464.7	8.8	1:0.011

Supplementary Table 6. EXAFS data fitting results at the Cu K-edge for Cu foil, Pd K-edge for Pd foil, Cu K-edge and Pd K-edge for $CuPd_{0.007}$ and $CuPd_{0.011}$.

**Sample**	**Absorption edge**	**Shell**	**R (Å)**	**CN**	**ΔE_0 (eV)**	**σ^2 ($\times 10^{-3}$ Å^2)**
Cu foil	Cu K	Cu-Cu	2.54	12	4.52	8.63
$CuPd_{0.007}$	Cu K	Cu-Cu	2.55	11.6	4.60	8.16
	Pd K	Pd-Cu	2.58	7.4	-1.34	5.20
$CuPd_{0.011}$	Cu K	Cu-Cu	2.54	11.9	4.76	8.39
	Pd K	Pd-Cu	2.57	5.7	0.34	5.49
		Pd-Pd	2.61	2.8	0.34	12.0
Pd foil	Pd K	Pd-Pd	2.74	12	5.62	4.59

Supplementary Table 7. A summary of Faradaic efficiencies for all products at different Cu catalysts.

Catalyst	Potential (V vs. RHE)	Faradaic Efficiency (%)					
		Hydrogen	Ethylene	Acetate	Ethanol	1-Propanol	Total
Cu	-0.49	17.1±2	49.3±2	5.9±0.5	11.6±2	13.8±0.5	97.7
	-0.52	13.8±1.1	50.5±1.4	5.6±0.3	13.7±1.5	10.7±1	94.3
	-0.55	10.4±0.5	51.5±0.7	6.1±0.2	14.3±0.5	10.1±0.2	92.4
	-0.58	8.5±0.3	52.8±1	8.7±1.2	15.8±1.2	9±1.2	94.8
	-0.60	8±0.2	51.4±2.5	8.1±2	17.4±1	8.6±2	93.5
	-0.62	7.8±0.5	52.9±3	9.2±1.5	17.8±1.2	8.4±0.5	96.1
	-0.64	7.3±0.7	53.4±2	9.8±1	18.6±1.2	7.8±0.3	96.9
	-0.66	6.9±0.3	54.8±1.4	9±1.3	20.1±0.8	6.9±0.6	97.7
	-0.69	6.5±0.4	55.5±2.2	11.2±2.1	19.8±2.5	5.4±0.5	98.4
	-0.71	7.5±0.8	52.3±1.8	14.4±1.8	17.7±2	3.1±0.3	95
$CuPd_{0.004}$	-0.47	16.3±2	33.2±2.3	8.1±0.5	17.8±1.2	13.6±1	89
	-0.51	12.6±0.5	37.7±1.5	9.2±1.5	21.8±0.6	9.5±0.5	90.8
	-0.54	11.1±1	39.5±0.7	11.5±2	23.5±1.1	9.7±0.7	95.3
	-0.57	10±1.2	40.8±2	11.5±1.2	25.9±2	8.8±0.2	97
	-0.59	9.3±0.5	41.8±2.5	9.1±1	26.3±1.4	8.1±0.5	94.6
	-0.61	9±0.3	43±1.3	10.6±0.7	28±1	7.2±0.3	97.8
	-0.63	8.9±0.2	43.9±0.5	11.6±1.3	28.5±1.2	6.9±1	99.8
	-0.65	9.2±0.5	44.4±3	12.7±1	27.4±0.5	5.6±0.4	99.3
	-0.68	9.9±1	43.7±3.2	11.4±1.4	26.2±1.2	4.8±0.2	96
	-0.71	11.9±1.5	42.2±2.1	11.4±1.5	26.1±1	4.6±0.2	96.2
$CuPd_{0.007}$	-0.49	14.6±2	33±1.2	7.7±1.2	23.3±1.5	14.3±2	92.9
	-0.52	12±1.2	36.2±0.5	11.3±2	25.9±0.7	10.3±1.5	95.7
	-0.54	10.6±0.5	36.2±1	12.9±0.5	27.1±1.3	8.7±0.3	95.5
	-0.56	9.2±0.8	36.6±0.6	13.1±1.3	28.7±1.2	7.6±0.2	95.2
	-0.58	9.2±1	36.9±0.4	13.9±1.1	30.1±2.1	7.4±0.3	97.5
	-0.60	8.8±0.4	36.7±0.2	14.5±0.4	31.5±0.8	6.6±0.5	98.1
	-0.62	8.5±2.5	37±1	14.9±1.2	33.2±1	6.3±0.2	99.9
	-0.64	9.5±2	36.8±0.8	18.6±1.3	29.2±1.2	5.3±0.7	99.4
	-0.67	11.6±3.2	34.6±1.5	19.2±0.7	26.1±2	4±0.3	95.5
	-0.72	19±3.5	29.5±2	21±2.1	22.4±1.5	3.3±0.2	95.2
$CuPd_{0.011}$	-0.51	12.8±0.5	35.1±2.3	5.7±0.5	21.2±1.2	8.4±1	83.2
	-0.53	11.5±1	35.2±1.2	8.2±1.2	25.8±1.5	5.1±0.5	85.8
	-0.58	11.6±0.2	36±2.5	11.7±2.3	25.1±1.5	4.2±1.3	88.6
	-0.60	12.7±1.2	34.8±3.2	14.4±1.4	23.7±1	2.7±0.4	88.3
	-0.65	21.3±2.3	28±4	19.2±1.2	21.4±0.7	1.5±0.5	91.4
	-0.72	29.6±3	20.3±2.3	19.5±2	16.3±2.3	0.6±0.2	86.3

Supplementary Table 8. Summary of alcohols electroproduction from CO_2/CO reduction tested at operating current densities > 100 mA cm^{-2}.

Catalyst	Reaction and cell type	Electrolyte	$FE_{alcohols}$ (ethanol + propanol, %)	$J_{alcohols}$ (ethanol + propanol, mA cm^{-2})	Potential (V vs. RHE)	Reference
**Pd-doped Cu**	**CORR in flow cell**	**1 M KOH**	**40**	**277**	**-0.62**	**This work**
**Pt-doped Cu**	**CORR in flow cell**	**1 M KOH**	**36**	**250**	**-0.63**	
OD-Cu	CORR in flow cell	1 M KOH	32	83	-0.60	Ref. [4]
Cavity-Cu	CORR in flow cell	1 M KOH	28	45	-1.36	Ref. [5]
Cu:molecule	CO_2RR in flow cell	1 M $KHCO_3$	41	124	-0.81	Ref. [6]
$Ce(OH)_x$/Cu/PTFE	CO_2RR in flow cell	1 M KOH	43	129	-0.70	Ref. [7]
Bimetallic Ag/Cu	CO_2RR in flow cell	1 M KOH	41	103	-0.67	Ref. [8]
Core-shell Cu_2S-Cu	CO_2RR in flow cell	1 M KOH	32	126	-0.92	Ref. [9]
CuAg alloy	CO_2RR in flow cell	1 M KOH	29	65	-0.64	Ref. [10]

Supplementary Notes

Supplementary Note 1

Reaction free energy calculation

The hydrogenation of HOCCH at the Cu surface using either a pre-adsorbed H or H from H_2O was simulated according to **Supplementary Equation 1** or **2**:

$$*HOCCH + *H \rightarrow *HOCHCH + * \qquad (1)$$

$$*HOCCH + H_2O + e^- \rightarrow *HOCHCH + OH^- \qquad (2)$$

where the * represent the adsorption site. Similarly, the dehydroxylation process was simulated by **Supplementary Equation 3**:

$$*HOCCH + e^- \rightarrow *CCH + OH^- \qquad (3)$$

the adsorption of H on different surfaces was calculated based on **Supplementary Equation 4**:

$$H_2O + * + e^- \rightarrow *H + OH^- \quad (4)$$

For each reaction, the free energies were given by **Supplementary Equation 5**:

$$\Delta G = \Delta E + \Delta ZPE - T\Delta S \qquad (5)$$

where ΔE is the reaction energy obtained by the difference between reactant and product molecules absorbed on catalyst surface; ΔZPE and ΔS are the change in zero point energy and entropy for each reaction. Meanwhile, entropy values of gaseous molecules are taken from the standard database in the NIST web-book[11], while the entropies of adsorbate and adsorption site are negligible. The zero-point energy for each adsorbate and free molecules can be obtained from the vibration frequency calculation, while the zero-point energy of adsorption site is negligible.

Supplementary Note 2

Surface energy calculation

The surface energies of various configurations, shown in **Supplementary Fig. 4**, were defined as **Supplementary Equation 6**:

$$\gamma = \frac{1}{A}(E_{slab} - E_{bulk}) - \gamma_{Cu} \qquad (6)$$

where γ is the surface energy of modified Cu configuration, A is the surface area of slab model, E_{slab} and E_{bulk} represent the total energy of slab and bulk model with same component. Here we only doped Pd on one surface of the periodic slab model, hence the surface energy of the modified surface should be obtained by subtracting the surface energy of unchanged surface, γ_{Cu}, which could be obtained by **Supplementary Equation 7**:

$$\gamma = \frac{1}{2A}(E_{slab} - E_{bulk}) \qquad (7)$$

Supplementary References

1 Xiao, H., Cheng, T. & Goddard III, W. A. Atomistic mechanisms underlying selectivities in C_1 and C_2 products from electrochemical reduction of CO on Cu (111). *J. Am. Chem. Soc.* **139**, 130-136 (2017).

2 Cheng, T., Xiao, H. & Goddard, W. A. Full atomistic reaction mechanism with kinetics for CO reduction on Cu (100) from ab initio molecular dynamics free-energy calculations at 298 K. *Proc. Natl. Acad. Sci. U. S. A.* **114**, 1795-1800 (2017).

3 Li, C. W., Ciston, J. & Kanan, M. W. Electroreduction of carbon monoxide to liquid fuel on oxide-derived nanocrystalline copper. *Nature* **508**, 504-507 (2014).

4 Jouny, M., Luc, W. & Jiao, F. High-rate electroreduction of carbon monoxide to multi-carbon products. *Nat. Catal.* **1**, 748-755 (2018).

5 Zhuang, T. T. *et al.* Copper nanocavities confine intermediates for efficient electrosynthesis of C_3 alcohol fuels from carbon monoxide. *Nat. Catal*. **1**, 946-951 (2018).

6 Li, F. W., *et al.* Cooperative CO_2-to-ethanol conversion via enriched intermediates at molecule–metal catalyst interfaces. *Nat. Catal.* **3**, 75-82 (2020).

7 Luo, M. C., *et al.* Hydroxide promotes carbon dioxide electroreduction to ethanol on copper via tuning of adsorbed hydrogen. *Nat. Commun.* **10**, 5814 (2019).

8 Li, Y. C., *et al.* Binding site diversity promotes CO_2 electroreduction to ethanol. *J. Am. Chem. Soc.* **141**, 8584-8591 (2019).

9 Zhuang, T. T. *et al.* Steering post-C–C coupling selectivity enables high efficiency electroreduction of carbon dioxide to multi-carbon alcohols. *Nat. Catal*. **1**, 421-428 (2018).

10 Hoang, T. T. H. *et al.* Nanoporous copper silver alloys by additive-controlled electrodeposition for the selective electroreduction of CO_2 to ethylene and ethanol. *J. Am. Chem. Soc.* **140**, 5791-5797 (2018).

11 Chase, M. W. NIST-JANAF thermochemical tables for oxygen fluorides. *J. Phys. Chem. Ref. Data* **25**, 551-603 (1996).
